# Evaluation of In Vitro Effectiveness of Seven Disinfectants over Controlling Candida on Complete Dentures

**Published:** 2012-02-01

**Authors:** A A Jafari, A Falah-Tafti, M H Lotfi-Kamran, H Fallahzadeh, F Akaberi

**Affiliations:** 1 Department of Parasitology and Mycology, Medical School, Shahid Sadoughi University of Medical Sciences, Yazd, Iran; 2 Department of Prosthodontics, Dentistry School, Shahid Sadoughi University of Medical Sciences, Yazd, Iran; 3 Department of Biostatistics and Epidemiology, School of Public Health, Shahid Sadoughi University of Medical Sciences, Yazd, Iran; 4 Dentist, Shahid Sadoughi University of Medical Sciences, Yazd, Iran

**Keywords:** In vitro, Candida, Disinfectant, Denture

Dear Editor,

Despite major advances in dentistry and dental prostheses, complete removable dentures are still widely used for oral rehabilitation of edentulous people. Denture surfaces prepare a favorable environment for adherence and proliferation of oral endogenous microorganisms, which is a crucial first step in the initiation of biofilm formation, caused denture stomatitis.[1] Candida species are known as the predominant oral etiologic agent of denture stomatitis, a common mucosal inflammation usually seen on the denturebearing mucosa of denture wearers.[2][3] There are several physical and chemical methods for cleaning of denture plaques.[4] Overnight soaking of denture in chemical solutions' including denture cleansers and detergents is an applicable and inexpensive method for improving of denture hygiene.[5] Ideal denture cleansers and disinfectants should be bactericidal, fungicidal, nontoxic and harmless to denture’s structure, effective for removing of organic and inorganic deposits on the denture.[4]

To compare the effectiveness of seven commercially available disinfectants for cleaning of complete denture, we performed an experimental study in Yazd Dentistry School. Sixty three complete maxillary dentures, which were used more than one year, were randomly divided to nine groups. Cultures from their internal surface were obtained by vigorous rubbing with a sterile cotton swab onto Sabouraud dextrose agar plates, incubated 48 hours at 30ºC for quantification of isolated Candida colonies. Isolated Candida colonies were also identified using germ tube test. Denture were then immersed for 1 hour in sterile ‘ziplock’ bags filled with 200 ml of sodium hypochlorite (2%, 1% and 0.5%), 0.2% chlorhexidine, 4% benzalconium chloride, 1% deconex and normal saline. Sterile distilled water and 100,000 IU solution of nystatin were also used as negative and positive controls in the current study. After the disinfectant protocol, the dentures were washed with sterile distilled water, re-swabbed and cultured as previously described before the disinfection. All cultures were performed by a single operator using sterile gloves, which were disposed and replaced for each denture. Density of isolated Candida colonies before and after disinfection was compared with Wilcoxon statistical (P<0.05) test using SPSS software.

All dentures were positive for Candida species in the initial culture before disinfection protocol (Table 1). Candida albicans was the most common isolated Candida species isolated from 71% of dentures, followed by non-albicans Candida species, isolated from 23% of dentures and 6% had mix species. All immersion solutions were found to reduce the growth of Candida in comparing with the initial culture. Two percent sodium hypochlorite (p=0.008), 1% sodium hypochlorite (p=0.016), 0.2% cholorhexidine (p=0.025) and 1% deconex (p=0.046) were known as the most effective disinfectants shown the maximum reduction in the load of isolated Candida species in current study (Table 2).

**Table 1:** Positive cultures of Candida on dentures before and after protocol, the numbers indicate the positive growth from total of seven dentures

**Disinfectant solutions**	**Before**	**After**
2% Sodium hypochlorite	7	0
1% Sodium hypochlorite	7	1
0.5% Sodium hypochlorite	7	1
02% Cholorhexidine	7	2
1% Deconex	7	2
4% Benzalconium chloride	7	3
Normal saline	7	4
Nystatine	7	0
Distilled water	7	7

**Table 2:** Load of Candida colonies (CFU) on the dentures

**Disinfectant solutions**	**Initial culture Mean (SD)**	**After the protocol Mean (SD)**	***P* value**
2% Sodium hypochlorite	202 (45.2)	3 (1)	0.008
1% Sodium hypochlorite	75.3 (34.8)	4.1 (2)	0.016
0.5% Sodium hypochlorite	119 (39.5)	6.2 (2.3)	0.036
02% Cholorhexidine	152 (48.3)	5.5 (2)	0.025
1% Deconex	89.2 (27)	11 (3)	0.046
4% Benzalconium chloride	109.3 (51.2)	15 (3.2)	0.066
Normal saline	121.7 (32.9)	29 (5.7)	0.083
Nystatine	155 (51.2)	0	0.0001
Distilled water	68.5 (8)	43.2 (18)	0.45

The results of the current study showed that all the initial cultures obtained from dentures, which taken directly from the patients’ mouth were positive for Candida species as expected. The objective of denture disinfection by immersing in chemicals is to obtain a clean, decontaminated prosthesis by removing the oral microbial contamination.[5] There are many studies, which showed more efficacy of chemical than physical methods.[6] The 2% and 1% sodium hypochlorite showed the highest cleaning effect followed by 0.2% chlorhexidine in the current study as also reported by Montagner et al.,[7] Furthermore 0.5% sodium hypochlorite, deconex (1%) and 4% benzalconium chloride showed a lower effect in disinfection of denture in the current study. Yilmaz et al. used 5.25% and 2% sodium hypochlorite as well as deconex and salvex and showed the same results;[8] however 5% sodium hypochlorite can damage dentures[9] and was not used in the present study.
